# Insect pest management in the age of synthetic biology

**DOI:** 10.1111/pbi.13685

**Published:** 2021-09-02

**Authors:** Rubén Mateos Fernández, Marko Petek, Iryna Gerasymenko, Mojca Juteršek, Špela Baebler, Kalyani Kallam, Elena Moreno Giménez, Janine Gondolf, Alfred Nordmann, Kristina Gruden, Diego Orzaez, Nicola J Patron

**Affiliations:** ^1^ Institute for Plant Molecular and Cell Biology (IBMCP) UPV‐CSIC Valencia Spain; ^2^ Department of Biotechnology and Systems Biology National Institute of Biology Ljubljana Slovenia; ^3^ Plant Biotechnology and Metabolic Engineering Technische Universität Darmstadt Darmstadt Germany; ^4^ Jožef Stefan International Postgraduate School Ljubljana Slovenia; ^5^ Earlham Institute Norwich Research Park Norfolk UK; ^6^ Institut für Philosophie Technische Universität Darmstadt Darmstadt Germany

**Keywords:** crop protection, insect pests, pheromones, biotechnology, synthetic biology

## Abstract

Arthropod crop pests are responsible for 20% of global annual crop losses, a figure predicted to increase in a changing climate where the ranges of numerous species are projected to expand. At the same time, many insect species are beneficial, acting as pollinators and predators of pest species. For thousands of years, humans have used increasingly sophisticated chemical formulations to control insect pests but, as the scale of agriculture expanded to meet the needs of the global population, concerns about the negative impacts of agricultural practices on biodiversity have grown. While biological solutions, such as biological control agents and pheromones, have previously had relatively minor roles in pest management, biotechnology has opened the door to numerous new approaches for controlling insect pests. In this review, we look at how advances in synthetic biology and biotechnology are providing new options for pest control. We discuss emerging technologies for engineering resistant crops and insect populations and examine advances in biomanufacturing that are enabling the production of new products for pest control.

## Introduction

It has been estimated that food production needs to increase by at least 50% over 2005 yields to meet the needs of the 2050 global population (Alexandratos, 2012). Improving crop yields and minimizing pre‐ and post‐harvest losses are critical to achieving these goals. While many factors from cultivation practices to climate events and outbreaks of pathogens are influential, arthropod pests account for around 20% of global annual crop losses, valued at over US$ 470 billion (Culliney, 2014). In developing countries, where population growth is most likely to outstrip increases in yields, pre‐harvest losses of grain crops from arthropod pests range from 15 to 100% and post‐harvest losses from 10% to 60% (Mihale *et al*., 2009). Alarmingly, a warmer climate is expected to alter agriculturally relevant characteristics of many insect pests, leading yield losses to increase by an estimated 10%–25% (Deutsch *et al*., 2018). In addition to direct damage, insect pests can also either directly transmit or facilitate secondary infections by viruses, bacteria and fungi. For example, the leafhopper, *Scaphoideus titanus*, transmits the grapevine yellows phytoplasma (Constable and Bertaccini, 2017), and the English grain aphid, *Macrosiphum avenae*, is a vector for barley yellow dwarf virus with infestations also leading to colonization by sooty moulds that establish on honeydew secretions (Fiebig *et al*., 2004).

Humans have applied chemicals with the aim of controlling insect pests for thousands of years. The use of elements such as sulphur as well as heavy metals and salts in agriculture was recorded by the Romans and other ancient civilizations. However, it was in the late 19th and early 20th century that advances in chemistry gave rise to synthetic pesticides. The first generation of insecticides consisted of highly toxic compounds, including the arsenic‐containing ‘Paris Green’, a copper acetoarsenite widely used in the United States between 1867 and 1900 (Hughes *et al*., 2011). Inorganic pesticides were largely replaced in the 1940s by a new generation of synthetic organic compounds, and, subsequently, production has boomed resulting in the marketing of numerous insecticides and general pesticides. Soon after their introduction, concerns arose about the effects of pesticides on the health of farmworkers and consumers as well as their impact on ecosystems (Oerke and Dehne, 2004). However, pesticide use increased throughout the twentieth century.

In the 1990s, new biotechnologies enabled the production of genetically modified (GM) crops. The first example of a transgenic insect‐resistant plant was provided by the expression of a cowpea trypsin inhibitor in tobacco (Hilder *et al*., 1987), and in 1995, ‘NewLeaf’ potatoes expressing the Cry3A protein from *Bacillus thuringiensis* (Bt) became the first genetically modified crop sold by the Monsanto Company. Since then, the global area of biotech crops increased from 1.7 million hectares in 1996 to 191.7 million hectares in 2018 with close to half of this area planted with pest resistant Bt varieties (ISAAA, 2018). In some regions, the adoption rate of Bt crops has been phenomenal. For example, in India, adoption rates for Bt cotton have been at 95% since 2015. Further, the use of GM crops has led to localized reductions in chemical pesticide use. For example, adoption of Bt eggplant in India reduced chemical pesticide applications by half while driving a 42% increase in yield (Ahmed *et al*., 2019). Similarly, planting of Bt cotton in Australia reduced pesticide use by up to 85% (Knox *et al*., 2006). In these contexts, one can argue that biotechnology has been successful and has provided a clear advantage over chemical products. However, in some world regions, these crops remain controversial due to differing perspectives on genetic modification as well as concerns about the emergence of resistant insect populations. Additionally, while some biotechnology crops have developed as public goods (Ahmed *et al*., 2019), increased costs associated with the use of biotechnology limit the spread of these technologies in low‐income nations.

In recent years, advances in computational technologies and biotechnologies have provided several alternatives to synthetic chemicals and first‐generation GM plants. These approaches promise to provide more sustainable and durable solutions. In this review, we will assess the opportunities and challenges afforded by these new technologies, noting that, for a genuine breakthrough, biotechnological innovations need to ensure that agriculture is socially, economically and environmentally sustainable to serve societies that increasingly recognize the value of biodiversity and environment (Pellé and Reber, 2015; Sayer *et al*., 2021).

## New generations of insect‐resistant crops

In insect‐resistant Bt crops, insecticidal activity is provided by expression of genes encoding parasporal crystalline protoxins, commonly known as Cry proteins (Palma *et al*., 2014). Nine different Cry proteins (Cry1Ab, Cry1Ac, Cry1A.105, Cry1Fa, Cry2Ab, Cry3Bb, mCry3A, eCry3.1Ab and Cry34/35Ab) have been used as transgenes, but insect populations have begun to evolve resistance to all of them. Interestingly, however, this has not happened in all insect species (Tabashnik and Carrière, 2017). For example, even after more than 20 years of exposure to a Bt maize in Europe, resistance has not been reported in the major pest, *Sesamia nonagrioides* (Castañera *et al*., 2016). Regardless, it is generally predicted that crops expressing multiple toxins with different modes of action are likely to perform better and delay the development of resistance (Bravo and Soberón, 2008; Tabashnik and Carrière, 2017). This has primarily been achieved through transgene pyramiding (Zhao *et al*., 2003). For example, three genes, *Cry*1Ac, *Cry*2A and *Gna* (snowdrop lectin), were inserted into indica rice targeting three major pests, rice leaf folder, *Cnaphalocrocis medinalis*, yellow stemborer, *Scirpophaga incertulas*, and the brown planthopper, *Nilaparvata lugens* (Maqbool *et al*., 2001). Additionally, pyramiding *Cry* toxins with vegetative insecticidal protein 3A (Vip3Aa) from *B. thuringiensis* has also been shown to diminish the risk of resistance (Carrière *et al*., 2015).

Several sources of resistance to insects have also been found within plants themselves. Many plants have evolved specialized metabolites to deter herbivores and sap feeders. Several of these have been used as ingredients in insecticidal formulations applied as foliar sprays including nicotine from tobacco, capsaicin from chilli peppers, pyrethrin from chrysanthemum and azadirachtin isolated from the Neem tree (Benelli *et al*., 2017; Pavela, 2016). More recently, transgenic approaches have also been demonstrated. For example, monoterpene biosynthesis in tomato fruits improved resistance against larvae of the corn earworm, *Helicoverpa zea* (Gutensohn *et al*., 2014). Further, the biosynthetic pathway of the volatile sesquiterpene (*E*)‐β‐farnesene, a plant metabolite that has also been described to act as an alarm pheromone in aphids (Bowers *et al*., 1972), was transferred to *Arabidopsis thaliana* (Beale *et al*., 2006) and, subsequently, to wheat (Bruce *et al*., 2015). While aphids were repelled and foraging behaviour of a natural parasite increased in laboratory conditions, these results were not replicated in the field (Bruce *et al*., 2015). Overexpression of an endogenous terpene synthase in rice (Os*TPS46*) led to overproduction of (*E*)‐β‐farnesene and limonene, deterring feeding by the bird cherry‐oat aphid, *Rhopalosiphum padi* (Sun *et al*., 2017), and tobacco plants genetically engineered to produce (*E*)‐β‐farnesene synthase from peppermint showed decreased levels of aphid infestation (Yu *et al*., 2013). Overexpression of (*E*)‐β‐caryophyllene, another volatile sesquiterpene found in plants and emitted by some insects as a semiochemical (El‐Sayed, 2021), in *A. thaliana* repelled the vector of citrus disease Huanglongbing, *Diaphorina citri* (Alquézar *et al*., 2017) and expression of the same gene in sweet orange produced similar effects (Alquézar *et al*., 2021). Lectins, common in many plants, bind to carbohydrate structures in the midguts of phytophagous insects causing disruption of the digestive system. Lectin genes have been transferred into crops including wheat (Stoger *et al*., 1999) and maize (Wang *et al*., 2005) conferring some resistance to aphid pests. Protease inhibitors, which aim to inhibit insect digestive enzymes, have also been deployed using transgenic strategies with genes originating from plants (Duan *et al*., 1996), sea anemones (Outchkourov *et al*., 2003) and mushrooms (Šmid *et al*., 2013).

To initiate their defence cascades, plants have evolved resistance genes (R genes). Many R genes encode nucleotide‐binding site leucine‐rich repeat (NBS‐LRR) proteins, which recognize specific pests and pathogens. Transgenic methods can be used to stack multiple R genes from resistant landraces or wild relatives into susceptible varieties. This has been successfully deployed to confer resistance to pathogens including late blight in potatoes (Ghislain *et al*., 2019) and rust in wheat (Luo *et al*., 2021). There have been some successes deploying R genes for resistance to insects. For example, transgenic expression of *Mi*‐*1.2*, an NBS‐LRR protein from wild tomato in susceptible commercial cultivars, conferred resistance to potato aphids, *Macrosiphum euphorbiae* (Rossi *et al*., 1998; Vos *et al*., 1998). One limitation, however, is that NBS‐LRR *R* genes can be highly specific to biotypes, and thus, when *Mi*‐*1.2* was transferred to eggplant, it could not confer resistance to eggplant‐feeding biotypes of the same species (Goggin *et al*., 2006).

An alternative strategy for engineering genetic resistance is to remove genes that confer susceptibility to pathogens (S genes). Genome editing technologies, such as those derived from bacterial CRISPR systems, have been successfully applied in a wide range of crops and can be used to eliminate the accumulation of specific gene products either by deleting the gene or by introducing mis‐sense mutations into the gene of interest (Gao, 2021). For example, the production of rice plants with targeted mutations in the cytochrome P450 gene *CYP71A* that catalyses conversion of tryptamine to serotonin resulted in insect‐resistant plants, which lacked serotonin but accumulated increased levels of salicylic acid (Lu *et al*., 2018). In several global jurisdictions, gene‐edited crops can reach market with fewer regulatory hurdles than GM crops (Waltz, 2018). This may make genome editing approaches more attractive for the development of new‐generation insect‐resistant crops than plants with transgene stacks. However, there is not yet global consensus on regulation: the European Court of Justice decided that, according to the text of Directive 2001/18/EC, genome‐edited products should be regulated as GM (Court of Justice of the European Union, 2018a,b), although a recent scientific opinion from the European Food Safety Authority concluded that some genome editing techniques pose no more hazards than conventional plant breeding (Naegeli *et al*., 2020).

## RNA interference for pest control

A different approach has leveraged RNA interference (RNAi) mechanisms to provide plants with molecules capable of down‐regulating essential genes in the insects that feed on them.

**Figure 1:**
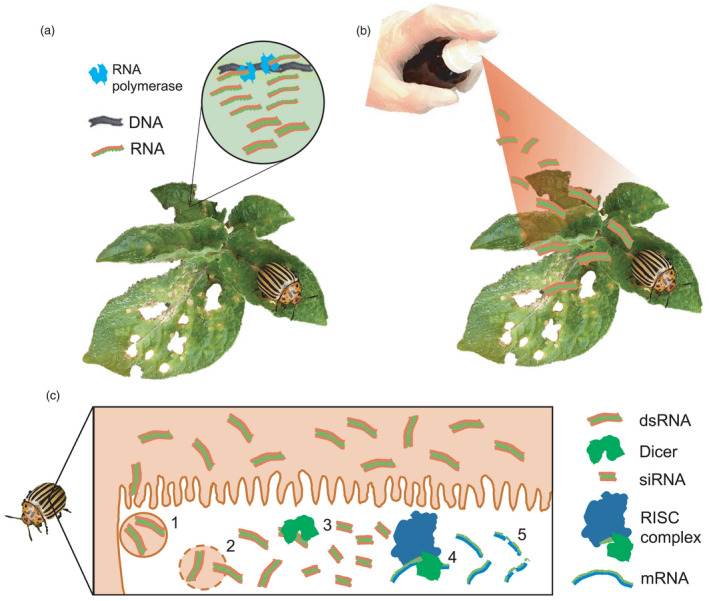
Methods for RNA interference (RNAi)‐mediated insect pest control. (a) In host‐induced gene silencing (HIGS), double‐stranded RNAs (dsRNA) are expressed from transgenes in the crop plant. (b) In spray‐induced gene silencing (SIGS), dsRNA is produced in a heterologous system and applied to the crop as spray before being consumed by the insect. (c) When insects feed on the plant, the dsRNA induces RNAi in the target species. Following uptake of dsRNAs into the insect gut epithelium cells by endocytosis (1) the dsRNAs are released, (2) short interfering RNA (siRNA) are generated by the Dicer complex (3) and the RNA‐induced silencing complex (RISC) mediates cleavage of target mRNAs with complementary to the siRNA’s leading strand (4). Finally, cleaved mRNAs are degraded by the nonsense‐mediated decay pathway (5).

RNAi is a regulatory mechanism present in many eukaryotes that uses double‐stranded RNA (dsRNA) to control the stability of mRNA (Figure 1). Since the discovery of the mechanism in the 1990s, the expression of engineered small interfering RNAs (siRNAs) has become a widely used approach for investigating gene function (Hannon, 2002). The first applications of RNAi technology to agricultural pest control were published over a decade ago (Baum *et al*., 2007; Mao *et al*., 2007). The most challenging part of this approach is to enable efficient uptake of dsRNA by the insect. Two options for the production and delivery of dsRNAs have been demonstrated. The first is the transgenic expression of dsRNAs from the crop genome, termed host‐induced gene silencing (HIGS, Figure 1a). In this approach, insects feeding on the crop will consume the dsRNA. In the second approach, dsRNAs are produced at high concentrations using a heterologous expression system and, following purification, are applied to insect‐infested crops as a foliar spray. This approach is termed spray‐induced gene silencing (SIGS, Figure 1b). In both approaches, in target species, the target genes will be silenced (Figure 1c) (Christiaens *et al*., 2020a).

Two studies published in 2007 provided proof‐of‐concept for the efficacy of RNAi‐based insect pest control. Mao *et al*. (2007) described a strategy to control the lepidopteran pest cotton bollworm, *Helicoverpa armigera*, by HIGS, engineering cotton plants to express dsRNAs targeting an insect gene encoding a cytochrome P450 that detoxifies the toxic plant metabolite, gossypol. Meanwhile, Baum *et al*. (2007) identified several target genes in western corn rootworms, *Diabrotica virgifera virgifera*, and Colorado potato beetles, *Leptinotarsa decemlineata*, of which RNAi‐induced silencing caused high mortality. Subsequent examples include the production of transgenic maize plants expressing a dsRNA targeting *Snf7*, an essential vacuolar sorting gene (Bolognesi *et al*., 2012). Recently, tomato plants expressing a dsRNA targeting a gene encoding a phenolic glucoside malonyltransferase, which detoxifies phenolic glycosides, showed complete resistance to the tobacco whitefly, *Bemisia tabaci*. Interestingly, the target gene was, itself, identified as being of plant origin, acquired by the insect in a horizontal gene transfer event (Xia *et al*., 2021).

Several studies have also inserted dsRNA expression cassettes into the chloroplast genome, a cellular compartment that lacks RNAi machinery. This strategy enabled transplastomic potato plants to be protected from herbivory by the Colorado potato beetle (Zhang *et al*., 2015) with subsequent studies showing the length of dsRNAs are a key factor influencing the strength of the insecticidal RNAi effect (He *et al*., 2020). Chloroplast‐expressed dsRNAs were also effective against the tobacco hornworm, *Manduca sexta* (Burke *et al*., 2019) and tobacco whitefly (Dong *et al*., 2020).

After more than a decade of research and development, RNAi‐based pest control products have advanced sufficiently to reach the market. A HIGS strategy for protection against western corn rootworm by Bayer Crop Science (formerly Monsanto) has been employed in MON87411 maize (Mat Jalaluddin *et al*., 2019). In general, the use of dsRNA provides several benefits over conventional insecticides, including the ability to control species specificity by targeting gene regions that vary between species. Other advantages include the rapid degradation of RNA into non‐toxic compounds and the lack of toxicity to vertebrates and other non‐target species. However, many challenges remain. The inherent lability of RNA, while ensuring that dsRNAs do not persist in the environment, is problematic in harsh environmental conditions and limits opportunities for SIGS approaches (Bramlett *et al*., 2020). Also, while some pests rapidly take up dsRNA resulting in high mortality rates, in other species low dsRNA uptake and nuclease degradation lead to inefficient results (Christiaens *et al*., 2020b; Shaffer, 2020). Success is also dependent on whether sufficient levels of dsRNA accumulate in the tissues on which the insects feed. Ongoing research is addressing dsRNA stability and uptake by the development of novel spray formulations, many utilizing nanomaterials (Christiaens *et al*., 2020b). Finally, while rapid increases in sequence availability improve the ability to design species‐specific dsRNAs, concerns remain about adverse effects on non‐target organisms (Rodrigues and Petrick, 2020) and significant questions remain about how insects may evolve resistance (Khajuria *et al*., 2018; Shaffer, 2020). Such concerns inform both regulatory and public deliberation on these approaches and may contribute to delaying or limiting their deployment in some world regions.

## Genetic methods for controlling insect populations

The use of genetic insect control methods dates to the first half of the twentieth century when impressive results were achieved by releasing radiation‐sterilized primary screwworms, *Cochliomyia hominivorax* (Scott *et al*., 2017). In this approach, known as the sterile insect technique (SIT), sterile males compete with the wild population to mate with females, which are then unable to produce viable offspring. However, SIT can be difficult as the fitness of irradiated males is generally compromised. In addition, it poses operational challenges including the need for the males to be separated prior to release to avoid crop damage by females and a risk that large numbers of non‐sterile insects may be unintentionally released from rearing facilities.

**Figure 2:**
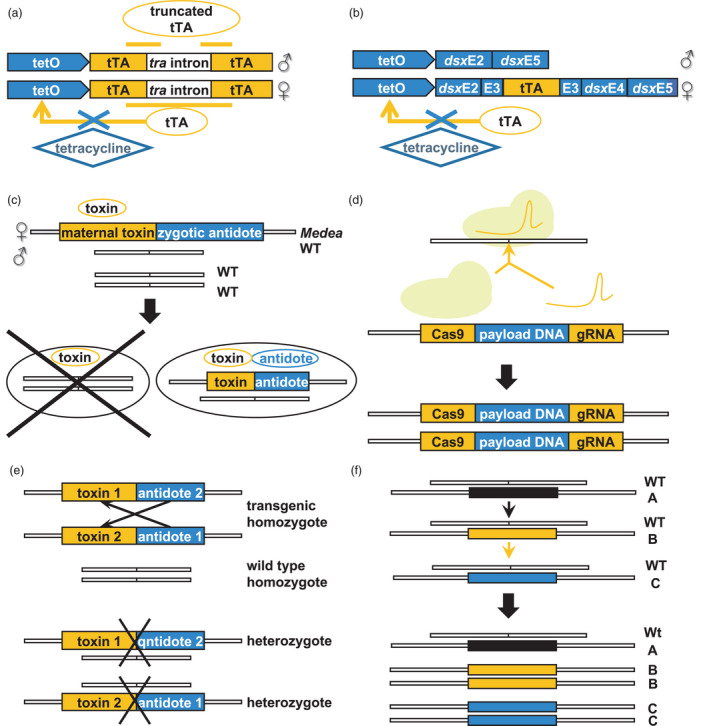
Strategies for genetic control of insect populations. (a and b): Release of insects carrying a female‐specific dominant lethal trait (RIDL). Expression of the transcription factor (tTA) is lethal and is controlled by the tetracycline‐repressible promoter (tetO) allowing transcription to be repressed with tetracycline. Female‐specific expression is conferred by (a) the inclusion of the *tra* (*transformer*) intron that is only spliced in females or (b) by placing the *tTA* gene within the female‐specific exon of the *dsx* (*doublesex*) gene. (c) Deployment of the maternal effect dominant embryonic arrest (*Medea*) gene drive facilitates expression of the maternal toxin during oogenesis in *Medea*‐bearing mothers. This is transmitted to all progeny, but *Medea*‐bearing embryos are rescued by expression of an antidote during embryogenesis. (d) In homing‐based gene drives, a transgene cassette is integrated at the locus of interest. The transgene encodes the Cas9/sgRNA genes to induce double‐stranded breaks and triggers homology‐directed repair in the equivalent locus. This ensures homozygosity of the transgene in any progeny and, thus, inheritance by all descendants. The use of underdominance or heterozygote inferiority results in a self‐limiting gene drive (e). In this case, heterozygotes have lower fitness than parental homozygotes. Homozygotes carrying two transgenic alleles survive because the toxin encoded by each allele is neutralized by the antidote encoded by the other allele. The spread of an underdominance gene drive requires high introduction rate and can be stopped by the release of wild type insects. In daisy‐chain gene drives (f) the drive and the elements they drive are located at independently segregating loci. In this example, A drives B, B drives C; A is not driven and declines. WT = wild type.

To overcome some of these issues, a strategy known as the release of insects carrying a dominant lethal trait (RIDL) was proposed. This typically involves the rearing and release of insects carrying a repressible dominant lethal transgene. To enable the rearing of such populations, sex‐specific conditional dominant lethality is employed. For example, the lethal gene is controlled using genetic elements that enable sex‐specific expression to be repressed by a specific diet component unavailable outside of the laboratory. This allows female insects to be selectively eliminated before release by withdrawing the repressor. A RIDL system was developed for *Drosophila melanogaster* using tetracycline‐repressible transcriptional trans‐activators under the control of female‐specific promoters (Thomas *et al*., 2000). Subsequently, a conserved sex determination mechanism based on alternative splicing (Figure 2a and b) was used as the basis for engineering female‐specific tetracycline‐repressible lethality in Mediterranean fruit flies, *Ceratitis capitata* (Fu *et al*., 2007), pink bollworms, *Pectinophora gossypiella* (Jin *et al*., 2013), and diamondback moths, *Plutella xylostella* (Jin *et al*., 2013). This has subsequently been marketed as Friendly™ technology by Oxitec Ltd. aimed at controlling insect vectors of human diseases as well as crop pests. Field‐cage studies with diamondback moths have demonstrated effectiveness in population suppression (Leftwich *et al*., 2014) and delays in the spread of insecticide resistance (Harvey‐Samuel *et al*., 2015). Further, in open‐field trials, dispersal, persistence and field survival rates of the released insects were shown to be similar to the wild type (Shelton *et al*., 2020).

More recently, gene drives have been proposed for facilitating the introduction of desirable traits into wild populations. Gene drives spread a desired allele into the population by enabling higher‐than‐Mendelian rates of inheritance. This approach can be employed for either population suppression or the modification of specific genes, for example, reversion of insecticide resistance alleles (Champer *et al*., 2016) (Figure 2a and d). A successful application of gene drives was the development of a maternal effect dominant embryonic arrest (*Medea)* in *Drosophila suzukii*, a highly invasive crop pest. This consisted of a miRNA expressed during oogenesis in Medea‐bearing mothers (toxin) that prevents the synthesis of a protein essential for embryogenesis together with a miRNA‐resistant copy of the target gene (antidote) expressed in Medea‐bearing embryos (Figure 2c; Buchman *et al*., 2018).

As molecular tools for targeted mutagenesis have become available, several different strategies for the construction and deployment of synthetic gene drives have been proposed (Esvelt *et al*., 2014; Hammond *et al*., 2016; Sinkins and Gould, 2006; Windbichler *et al*., 2011). CRISPR‐gene drives have been tested in *D. melanogaster* (Gantz *et al*., 2015) as well as in two species of *Anopheles* mosquitoes, vectors of the malaria parasite, suppressing of reproduction of *A. gambiae* (Hammond *et al*., 2016) and blocking parasite development in *A. stephensi* (Gantz *et al*., 2015). Symbiotic associations of insects with microorganisms can also be exploited for suppression of pest populations and for gene drives (Panagiotis and Bourtzis, 2007). For example, a so‐called incompatible insect technique (IIT), based on cytoplasmic incompatibility, induced by *Wolbachia* was used to control laboratory populations of the Mediterranean fruit fly (Zabalou *et al*., 2004).

Opinions on the application of genetic methods for pest insect control vary (Zhang *et al*., 2020). While SIT programmes using irradiated insects have previously faced little resistance, the release of genetically modified insects has encountered criticisms (Baltzegar *et al*., 2018). Concerns have been raised about the inherent pervasiveness and potentially irreversibility of gene drives, raising questions of proportionality (Callaway, 2018). Support for the use of gene drives has been found to be higher when used to control non‐native pest species, or if they include a mechanism to limit drive spread (Jones *et al*., 2019). Consequently, it is possible that the development of self‐limiting gene drive systems, for example underdominance (Dhole *et al*., 2018) or daisy‐chain drives (Noble *et al*., 2019) (Figure 2e and f), may provide options for wider deployment of these technologies in the future, particularly if directed towards invasive and non‐native pests.

## Biomanufacturing pest control products

As noted above, several natural products, particularly plant specialized metabolites, have been used as agents for insect pest control. While this is a viable option for abundant chemicals for which extraction from the original plant source is economically viable and for those that are easily produced via synthetic chemistry, the use of many bioactive organic products is limited by the inability to affordably scale up production. Biomanufacturing, accelerated by the application of synthetic biology approaches to metabolic engineering, is an emerging technology providing the potential for the large‐scale production of complex molecules with little or no chemical waste. Direct or foliar application of biomanufactured molecules, either purified from their production organisms or used as semi‐processed formulations, may encounter fewer regulatory barriers than engineered crops.

To date, the most significant advances have been made using microbial organisms as chassis for biomanufacturing natural products for health and industry (Yang *et al*., 2020), including known insect repellents nootkatone (Guo *et al*., 2018) and cembratrienol (Zhang *et al*., 2021). However, plants have also been envisioned as platforms for bioproduction of insecticidal compounds. For example, the biosynthesis pathway of pyrethric acid, the monoterpene acyl moiety of the widely used pyrethrin insecticides, has been transferred to *Nicotiana benthamiana* (Xu *et al*., 2019). More excitingly, bioproduction has also been proposed as a potential solution for manufacturing novel classes of insect‐controlling chemicals including insect sex pheromones. These, being highly species‐specific, provide an attractive alternative to broad‐spectrum pesticides. Further, pheromones are typically volatile and emitted in minute quantities into the environment rather than being applied to the crop itself. Crucially, they are likely to remain effective against pesticide‐resistant insect populations; evolution of pheromone resistance is considered unlikely due to strong selection against losing the ability to find a mate (Rizvi *et al*., 2021). While several pheromones are easy to produce by chemical synthesis and have already reached market, the chemical synthesis of many other insect sex pheromones remains challenging or expensive, mainly due to their complex molecular structures and requirement for multiple stereoselective steps. Thus, production costs can range from several hundred to several thousand dollars per kilogram, a cost only affordable for the protection of high‐value agricultural products such as speciality fruits (Petkevicius *et al*., 2020). Even when affordable chemical synthesis is possible, biomanufacturing may represent a more sustainable method of production, requiring renewable feedstocks and producing little or no hazardous waste.

**Figure 3:**
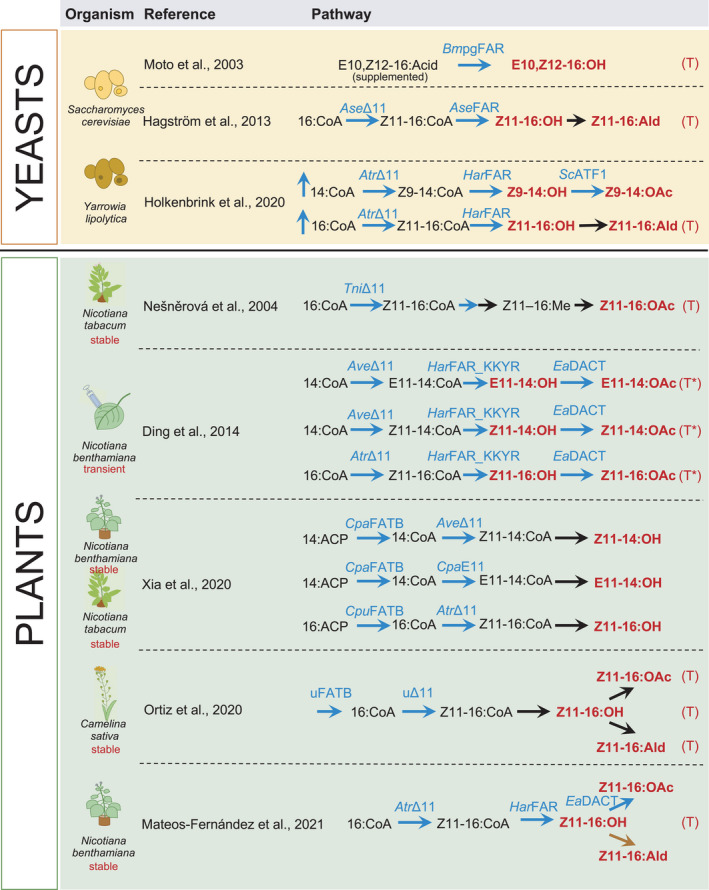
Strategies for the biosynthesis of lepidopteran sex pheromones. Blue arrows represent synthesis by a heterologous enzyme; black arrows represent chemical synthesis; purple arrows represent synthesis by an endogenous enzyme. Metabolites in red text are known to act as sex pheromones. (T) indicates experimental assays have shown that the molecule affected the behaviour of moths and (T*) indicates that the behaviour assay was conducted with a chemically synthesized molecule, even if also produced via biosynthesis. *Bmp*gFAR *= Bombyx mori pheromone gland fatty‐acyl reductase*; *Ase*Δ11 *= Agrotis segetum* Δ11 fatty‐acyl desaturase; *Ase*FAR = *Agrotis segetum* fatty‐acyl reductase; *Atr*Δ11 *= Amyelois transitella* Δ11 fatty‐acyl desaturase; *Ha*rFAR *= Helicoverpa armigera* fatty‐acyl reductase; *Sc*ATF1 = *Saccharomyces cerevisiae* alcohol acetyl transferase; *Trichoplusia ni* Δ11 fatty‐acyl desaturase; *Ave*Δ11 *= Argyrotaenia velutinana* Δ11 fatty‐acyl desaturase; *Ha*rFAR_KKYR *= Helicoverpa armigera* fatty‐acyl reductase with C‐terminal endoplasmic reticulum retention signal; *Ea*DACT *= Euonymus alatus* acetyltransferase; *Cpa*FATB *Cuphea palustris* 14:ACP fatty acid thioesterase; *CpuFATB = Cuphea pulcherrima* fatty acid thioesterase; *Cpa*E11 = *Choristoneura parallela* E11 desaturase; FATB = unspecified acyl carrier protein thioesterase; uΔ11 = unspecified Δ11 fatty‐acyl desaturase.

Lepidopteran sex pheromones, mainly fatty alcohols and their corresponding aldehyde and acetate derivatives, are some of the best‐studied pheromones due to the economic impact as well as their relatively simple chemical formulation. Biosynthesis of bioactive lepidopteran sex pheromone and chemically modifiable precursors has been explored in several biological chassis (Figure 3). Hagström *et al*., (2013) demonstrated production of the fatty alcohol components of moth sex pheromones in *Saccharomyces cerevisia*e expressing insect fatty‐acyl desaturases and reductases. Another yeast, the oleaginous *Yarrowia lipolytica*, was additionally engineered to improve the yield of moth sex pheromones by down‐regulating fatty alcohol degradation and storage lipids besides expressing pheromone synthesis enzymes (Holkenbrink *et al*., 2020). Alternative approaches based on a combination of biological and chemical synthesis, such as a dried formulation of yeast biomass that is processed into desaturated fatty alcohols, have also been explored and patented (Konrad *et al*., 2017). Recently, BioPhero, a spin‐out from the Technical University of Denmark, demonstrated large‐scale production of field‐tested moth pheromones in yeast (Petkevicius *et al*., 2020).

Plants are also being used to trial the production of insect sex pheromones due to their high biomass and ability to produce complex molecules. The first example of plant production of insect sex pheromones described a transgenic tobacco line producing moth sex pheromone precursors (Nešněrová *et al*., 2004). Subsequent examples demonstrated the production of precursors in *Nicotiana spp*. (Xia *et al*., 2020) and in false flax, *Camelina sativa*, where products accumulated in seeds (Ortiz *et al*., 2020). In 2014, several fatty alcohols and their corresponding aldehyde and acetate forms were produced via transient expression (agroinfiltration) of *N*. *benthamiana* leaves, providing a biologically active pheromone blend (Ding *et al*., 2014).

Pheromone production in plants also provides the opportunity of direct dispensation from the living production platform, negating the need for extraction and packaging into man‐made traps or dispensers. While living biodispensers would not be useful for attract and kill strategies where the pheromone and an insecticide are combined within a trap, they could be used in mating disruption strategies in which the aim is to impede the ability of males to locate a female mate. In 2020, live plant dispensers of chemical signals were included in a shortlist of emerging products of bioengineering (Kemp *et al*., 2020) and, recently, transgenic *N. benthamiana* plants transformed with a three‐gene pathway resulted in the production of high levels of the *(Z)*‐11‐hexadecen‐1‐ol (Z11‐16OH) and *(Z)*‐11‐hexadecenyl acetate (Z11‐16OAc) components of moth pheromones (Mateos‐Fernández *et al*., 2021). Although pheromone production was shown to negatively influence plant growth and only a small fraction of the pheromones were released to the environment in volatile forms, this first report of a living biodispenser could pave the way for future iterations.

As discussed above, transgenic plants expressing the insect alarm pheromones (*E*)‐β‐farnesene and (*E*)‐β‐caryophyllene showed decreased levels of insect infestation. While work to date has aimed at engineering these pathways into crop plants, these compounds might also be targets for biomanufacturing, enabling their inclusion in sophisticated chemical blends. Of further interest is the ability to produce blends that include or co‐emit plant‐based compounds known to play roles in insect communication. Some plant endogenous volatiles have been described to act synergistically with insect sex pheromones to increase their attractiveness (Bruce and Pickett, 2011; Gregg *et al*., 2018). Several terpenoid and aromatic compounds exert this effect towards Lepidoptera (von Arx *et al*., 2012), while some green leaf volatiles (C6 fatty acid derivatives) emitted as a consequence of phytophagy is active towards a broad range of species (Dickens *et al*., 1993). Future insect sex pheromones strategies could consider including such compounds in novel chemical blends to optimize their effects. Further, such blends might be produced by co‐expressing the desired biosynthetic pathways within the same production chassis. This approach could include herbivore‐induced plant volatiles (HIPVs) to attract predators and parasitoids of herbivore pests (Nishida, 2014; Wei and Kang, 2011).

## Harnessing biocontrol

Biological pest control methods are now a crucial component of integrated pest management strategies. Natural predators, parasitoids, competitors and pathogens are all employed to control insect infestations. However, while progress has been made at improving biocontrol agents using artificial selection (Lirakis and Magalhães, 2019) including boosting thermotolerance of entomopathogenic fungus *Metarhizium anisopliae* (de Crecy *et al*., 2009) and selecting for truncated wings in predaceous ladybird beetles to prolong residence on aphid‐infested host plants (Lommen *et al*., 2019), to date, little has been done to exploit their full potential. Despite numerous experimental efforts and advances, aside from a few recombinant *Bt* strains, genetically engineered biocontrol agents have not been widely commercialized (Karabörklü *et al*., 2018). However, the application of emerging genetic and genomic approaches to biocontrol agents could become an integral part of developing efficient biocontrol strategies (Leung *et al*., 2020).

Entomopathogenic agents, including fungi, bacteria, viruses and nematodes, represent the majority of biocontrol agents on the market (Lacey *et al*., 2015). Biotechnology provides the opportunity to engineering these species to improve their efficacy. For example, transgenic approaches have been used to increase the efficacy of entomopathogenic fungi following overexpression of virulence factors, toxins and proteins that disrupt the homeostasis of physiological processes (Fan *et al*., 2012; St. Leger *et al*., 1996; Wang and St Leger, 2007). Additionally, fungi, bacteria and viruses have been engineered to produce insect‐specific dsRNAs (Al Baki *et al*., 2020; Bao *et al*., 2016; Mysore *et al*., 2019), and baculoviruses have been used as an effective expression and delivery system for insecticidal proteins and hold promise for implementation of RNAi (Harrison and Bonning, 2000; Kroemer *et al*., 2015). In the future, genetic engineering might also be applied to improve the resistance of entomopathogenic agents to abiotic stresses to modulate their host range or their sensitivity to chemical pesticides and fungicides (Bielza *et al*., 2020). However, to achieve this, a better understanding of virulence factors (Gao *et al*., 2020; Semenova *et al*., 2020), fitness (Mou *et al*., 2020) and host–pathogen interactions (Hussain, 2018; Yang *et al*., 2014a) is required.

Species currently known to negatively impact pest populations are also a useful source of molecules that could be leveraged using the transgenic and biomanufacturing technologies described in previous sections. Besides the well‐known *Bt* toxins, toxins from bacterial symbionts of entomopathogenic nematodes *Photorhabdus luminescens* and *Xenorhabdus nematophilus* (Ffrench‐Constant and Bowen, 2000) and from soil‐dwelling bacteria *Pseudomonas chlororaphis* (Schellenberger *et al*., 2016), as well as many other natural peptides (Harrison and Bonning, 2010; Paul and Das, 2021), have been proposed as potential insecticides. These have been shown to be effective following oral ingestion by insects either alone or following fusion to carrier proteins (Yang *et al*., 2014b). The use of specific bioactive molecules may also avoid the potential ecological effects of widespread release of entomopathogens.

## Conclusions and outlook

The ability to engineer organisms has steadily increased in the first decades of the 21^st^ century, driven both by the availability of genome engineering tools and by the application of engineering approaches (synthetic biology) to biotechnology that has been instrumental in enabling scalable biomanufacturing of natural products (Lorenzo *et al*., 2018). Interestingly, and with similarities to crop varieties produced by radiation, the release of a population of irradiated insects encountered little resistance in the 20^th^ century. However, while genetically modified insect populations show potential and populations of engineered diamondback moths, pink bollworms and, most recently, *Aedes aegypti* mosquitoes, have all been released in non‐contained trials in the United States, engineered populations, particularly using gene drives, seem less likely to have an easy route to market (Baltzegar *et al*., 2018).

In recent years, concerns about the ecological impacts of broad‐spectrum pesticides have increased, leading to bans and a growing interest in alternative methods of pest control (Butler, 2018). Evidence suggests that insect‐resistant crops have significantly reduced the use of pesticides (Ahmed *et al*., 2019; Knox *et al*., 2006; Wilson *et al*., 2018). However, the appearance of resistant populations has caused concern and is likely to influence the strategies used to develop new generations of insect‐resistant crops. Further, even where resistant populations have not emerged, the widespread adoption of insect‐resistant GM crops has had unforeseen consequences allowing pests other than the initial target species to flourish. For example, the planting of Bt cotton successfully reduced damage from bollworms and leaf‐feeding insect infestations, but sap feeders including whitefly, leafhoppers, aphids and thrips emerged as new dominant causes of crop loss (Shera *et al*., 2020). Examples such as this highlight the difficulties of pest management.

With limitations on broad‐spectrum chemicals, methods such as pheromones and RNAi, both capable of superb species specificity, may be increasingly valued. While pheromones have been in use for some decades, products available for agricultural pest control have been limited to those able to be chemically synthesized at low cost. Recent advances in synthetic biology and metabolic engineering are rapidly expanding the diversity of complex molecules that can be produced at scale providing new opportunities for pheromone production. This is exemplified by the recent emergence of pheromone biotechnology companies such as BioPhero and Provivi. With many nations outlining strategies to develop their bioeconomies, innovation in biomanufacturing that will increase the feasibility of commercial scale production looks set to increase (Kuckertz, 2020). Further, the application of synthetic biology to plant metabolic engineering provides the opportunity for novel deployment strategies such as living biodispensers (Kemp *et al*., 2020).

However, while biotechnology may provide multiple options for novel methods of pest control, many products will require regulatory systems that, for some products and in some regions, may not yet be in place. Additionally, they will require the support of growers and consumers for which open dialogues, including the potential of new technologies to contribute to social progress, will be beneficial (Bauer and Bogner, 2020).

## Conflict of interest

All authors agree to publication of the manuscript. The authors report no commercial or proprietary interest in any product or concept discussed in this article.

## Author contributions

NJP, DO, AN, SB and KG were involved in conceptualization and supervision. RMF, MP, IG, MJ, SB, KK, EMG, JG, AN and NJP drafted the text. All authors contributed to revising and editing the text.
